# The Role of Images on Illness Behaviour: Interdisciplinary Theory, Evidence, and Ideas

**DOI:** 10.1177/0033294120945602

**Published:** 2020-08-05

**Authors:** Jennifer Murray, Brian Williams

**Affiliations:** School of Health and Social Care, Edinburgh Napier University, Edinburgh, UK

**Keywords:** Visual intervention, visual communication, illness behaviour, theory informed intervention, theory based intervention

## Abstract

If illness behaviour is to be fully understood, the social and behavioural sciences must work together to understand the wider forms in which illness is experienced and communicated with individuals and society. The current paper synthesised literature across social and behavioural sciences exploring illness experience and communication through physical and mental images. It argues that images may have the capacity to embody and influence beliefs, emotions, and health outcomes. While four commonalities exist, facilitating understandings of illness behaviour across the fields (i.e., understanding the importance of the patient perspective; perception of the cause, sense of identity with the illness, consequences, and level of control; health beliefs influencing illness experience, behaviours, and outcomes; and understanding illness beliefs and experiences through an almost exclusive focus on the written or spoken word), we will focus on exploring the fourth commonality. The choice to focus on the role of images on illness behaviour is due to the proliferation of interventions using image-based approaches. While these novel approaches show merit, there is a scarcity of theoretical underpinnings and explorations into the ways in which these are developed and into how people perceive and understand their own illnesses using image representations. The current paper identified that the use of images can elucidate patient and practitioner understandings of illness, facilitate communication, and potentially influence illness behaviours. It further identified commonalities across the social and behavioural sciences to facilitate theory informed understandings of illness behaviour which could be applied to visual intervention development to improve health outcomes.

## Introduction

Illness behaviour can be defined as any behaviour undertaken by an individual to relieve an experience of illness or to better define the meaning of the illness experience (Kasl & Cobb, 1966; Mechanic, 1995). There has been an observable increase in the prevalence of chronic health conditions since 2001, with increases from 35% to 42% in prevalence being observed within general practice registration data between the years 2004–2011 (van Oostrom et al., 2016) and increases from 41% to 47% being seen within self-report data between the years 2001–2011 (van Oostrom et al., 2016). The World Health Organisation (World Health Organization, 2007) reported chronic diseases to be the leading causes of death and disability which account for close to 60% of global deaths, with this expected to rise to 73% by 2020. This increase in the prevalence of chronic conditions (van Oostrom et al., 2016) means that future attempts to improve health outcomes and illness experiences are likely to focus on individuals who are already symptomatic; thus, will focus on illness behaviour. This may include the interpretation and appraisal of symptoms, decisions to seek or delay health care, ongoing social and psychological coping strategies, adherence/concordance to recommended treatments and therapies, and the general ongoing uptake of available support services.

### Cross-disciplinary understandings of the role of images within illness behaviours

Social and behavioural sciences have contributed significant theory to our understandings of illness behaviours. In the context of the current paper, we adopt a broad definition of illness behaviour which is drawn from the behavioural medicine domain: illness behaviours are considered to be the observable actions or reactions to actual or perceived physical, emotional or mental illness; these can include self-care or the use of healthcare services (Rau & Williams, 2013).

An examination of the contributions across the social and behavioural sciences literatures reveals four similarities. First, although differing terms are used, each highlights the importance of the patient perspective through an emphasis on illness beliefs (e.g., sociology), explanatory models (e.g., anthropology) or illness representations (e.g., psychology). Second, despite varying theoretical and philosophical assumptions and underpinnings, empirical research in each discipline generally uncovers beliefs that categorise across similar dimensions. For example, within medical anthropology, Kleinman’s Kleinman (1987) ‘explanatory model’ encompasses beliefs about diagnosis, cause, and appropriate treatments (Helman, 1998; Kleinman, 1987); while, within health psychology the concept of “illness representations” points to the parallel issues of illness identity, cause, consequences, timeline and cure/control (Leventhal et al., 1980; Leventhal & Nerenz, 1985). A third similarity is the consistent evidence across these disciplines, particularly across psychology and medical sociology, indicating that health beliefs may influence illness experience (Bishop & Converse, 1986; Lobban et al., 2003; Scambler & Hopkins, 1986), illness behaviours (Horne & Weinman, 2002; Ross et al., 2004) and ultimately health outcome (Barsky et al., 2002; Petrie et al., 2007). A final commonality is the cross-discipline emphasis on understanding illness beliefs and experience through an almost exclusive focus on the written or spoken word.

While health messages are almost always communicated through verbal or text-based means, a growing body of evidence is emerging which suggests that these traditional delivery systems may not be the most effective (e.g., Garcia-Retamero & Galestic, 2010; Williams & Cameron, 2009). For example, patient perceptions of risk were found to improve and be less susceptible to framing effects on health information when a visual aid was used (Garcia-Retamero & Galestic, 2010). However, a recent systematic review exploring the efficacy of video-based visual health interventions found variations in the effectiveness of the interventions, with video interventions tailored to the individual or specific condition being more effective than non-tailored video interventions (Tuong et al., 2014). These findings suggest that while images have been used in health promotion campaigns in the same manner as in commercial advertisements, this is often carried out with little or no theoretical consideration in their use and effectiveness.

The current paper argues that if illness behaviour is to be more fully understood, academics across the social and behavioural sciences must acknowledge and encompass the wider forms in which illness is experienced and communicated in contemporary society. We suggest that illness may be experienced and communicated through physical and mental representations (i.e., mental images), and that these may have the capacity to embody and influence both beliefs and emotional sequalae, and thus an array of important illness behaviours and outcomes.

This understanding is imperative to academics, health professionals and others engaged in intervention development in healthcare. In recent years there has been an increase in the use of visual communication at both the societal and population level (Williams & Cameron, 2009) and within healthcare interventions (Murray et al., 2001, 2016; Williams et al., 2012) to convey complex information, influence beliefs and emotions (Williams & Cameron, 2009) and, ultimately, elicit a change in health behaviour. However, at present, the evidence base applied to the development of visual interventions is scant (Williams & Cameron, 2009). This poses a risk to the quality of the interventions themselves and, more importantly, to the messages conveyed to and understandings of patients (Williams & Cameron, 2009).

The current paper therefore aims to review the social and behavioural sciences literature to explore what is known about the role of visual stimuli and images on illness behaviours. The main literatures included within this review are decision sciences, health and behavioural psychology, and medical sociology. Wider literatures and papers are also included but with a less detailed focus than the former.

The current paper will explore theoretical and empirical evidence across the social and behavioural sciences examining the importance of images in illness behaviours. It will discuss some of the dominant health behaviour models relevant to the area, and then examine possible ‘precognitive’ or ‘unconscious’ influences of images on judgement and decision making and illness behaviours. The role of image changes will be discussed in relation to behaviour change and non-change, and how these are determined depending upon the role of symptom interpretation and emotional activation. The impact that this has on health outcomes and health care will then be discussed. Prior to exploring the theoretical and empirical evidence, however, it is important to identify what we mean by “image” within the current paper. This will now be defined.

### What do we mean by “image”?

For the purposes of this paper, “image” is defined in a broad sense to include any visually based or mental representation or embodiment. This definition reflects an essentially phenomenological stance that focuses on a principal “sense”. However, given ongoing research and debates within neuropsychology, and the evident role of a visual dimension within classic illness metaphors (Helman, 1985; Sontag, 1978, 2001), we take the “visual” to not only encompass physical sight, but also mental representations of images. We will primarily use ‘image’ in the remainder of the current paper to maintain consistency in the terminology used. However, where appropriate and better fitting with the papers or theories being discussed, we may use the term used in the original paper/theory (e.g., visual stimuli, mental representation).

## Theoretical and empirical evidence to support the importance of images

There is growing theoretical and empirical evidence to indicate that images may influence the experience of illness, illness behaviour and health outcome (e.g., Humphris & Williams, 2014; Murray et al., 2001, 2013, 2016; Styles et al., 2013; Thorne et al., 2005; Williams & Cameron, 2009). From a psychological perspective, it is known that both cognition and emotion, and their corresponding interplay, can influence behaviour. Indeed, the central components of the prime theoretical model at the heart of psychological research into illness behaviour, Leventhal’s Common Sense Model, which is embedded within the Self-Regulatory Model of Health Behaviour (Leventhal et al., 1980, 2003), suggests that symptom perception leads to two parallel processes: one relating to cognition (in particular, the beliefs along five dimensions of illness representations highlighted earlier: illness identity; cause; consequences; timeline; cure/control); and one to emotion, and both of which feed into a coping strategy. More broadly, the Common Sense Model supports the understanding of people’s responses to illness, explaining the ways in which illness perceptions, incorporating knowledge and personal experiences, can influence coping strategies and health outcomes.

We suggest that there is evidence that physical and mental images may influence behaviour at four points within the Common Sense Model: a ‘precognitive’ influence on behaviour that bypasses cognition and emotion; a prompt that forces an individual to notice a deviation in normality and thus promote symptom perception; a means by which cognition is both embodied and influenced; and an impact directly on emotion. Each of these four points within the Common Sense Model will be discussed in turn within this section, with the relationship between images and illness behaviour being the focus.

### Precognitive influences on illness behaviours

Precognitive influences on illness behaviours may be defined as those influences which we are not necessarily aware of as having an impact on our judgements, decisions and illness-related behaviours (see, for example, Kahneman, 2011, for a thorough overview of unconscious and conscious decision making). These would not be influences that are consciously recognised by a person and may better be described as instinctual, automatic, intuitive, unconscious or heuristic influences. While it may be argued that all human experiences and influences on our behaviours, and hence our illness behaviours, must be cognitive to some degree, these precognitive influences may be best considered as those influences that affect our actions and beliefs that we are not consciously aware of. Note that the use of the term ‘precognitive’ in the current paper is not the same as those studies which posit that precognition is a predictor of future outcomes; we instead use the term to mean unconscious influences on our decision making, as described in this section.

When considering the complexity of influences on illness behaviours, the influence is not merely on behaviour, but also on cognitions such as judgements and decisions. To best understand the role of precognitive influences on illness behaviours, we will first summarise recent theory on human judgement and decision making and will follow this with consideration for precognitive influences on image interpretation and illness behaviours.

#### Precognitive influences on judgement and decision making

Models of human decision making (leading to behaviours), in particular dual process models, offer valuable insights to help us understand and define these precognitive influences on illness behaviour. Numerous decision making and judgement theories have been proposed. One recent theory was proposed by Kahneman (2011). In this dual process theory, it is suggested that we have two systems with which we make decisions and judgements. System 1 is ‘fast’ and allows us to make decisions quickly, using little cognitive effort by using: evolved cognitive heuristics (or ‘cognitive rules of thumb’; Murray & Thomson, 2010); intuitions which have been learned and engrained into our cognitive schema through past experiences; and/or emotions. We are largely unaware of these processes and so they could be argued to be precognitive, in the context of the current paper and the language used in the Common Sense Model discussed previously.

System 2 refers to those instances where our choices are determined through a slower, more cognitively taxing, rational cognitive process, where the decision maker must consciously consider and ‘weigh-up’ the pros and cons of a decision before taking action. System 1 and 2 exist and occur in tandem and some authors more recently have argued that the two exist on a continuum rather than as separate/distinct systems (i.e. existing as ‘quasirational’; Dhami & Thomson, 2012). However, all dual process theories stress that it is the faster, heuristic, System 1 which is used in the majority of our decisions. Thus, the exploration of the ‘precognitive’ influences on behaviour is both important and necessary for understanding illness-related behaviours. When discussing precognitive influences on illness behaviours, the current paper is framing these within an operational definition akin to the ‘System 1’ processes proposed by Kahneman (2011).

#### Precognitive influences on image interpretation and illness behaviours

Applying these theoretical arguments and definitions, there is evidence in regard to physical attraction and the avoidance of dangers that visual stimuli can influence behaviour precognitively. For example, facial symmetry is associated with both perceived beauty (Rhodes et al., 1998) and health (Rhodes et al., 2001) and is observed to be associated with human mate choice selection preferences (Scheib et al., 1999). Indeed, phenotypical symmetry is also associated with better health outcomes (*ibid*), indicating a complex interplay between these associations as the product of evolutionary processes and socio-cultural pressures to improve genetic transferral via mate choice selection leading to successful offspring (Rhodes et al., 1998). If this is the case, then visual stimuli may have a precognitive influence within health and illness behaviours more generally.

A clear and contemporary example in the context of health behaviours is the Western association between a ‘tanned’ skin colour, health and attractiveness (Broadstock et al., 1992) amongst Caucasians. In modern Western society, behaviours aimed at increasing UV exposure such as the use of sun-beds and other tanning pursuits that may be hazardous to health have increased (Leary & Jones, 1993). A converse association is seen more recently amongst African and South East Asian beauty trends, with skin whitening and bleaching products becoming popular, demonstrating that the socio-cultural construction of beauty standards can overcome natural evolve appearance preferences. Simply providing knowledge and education about the potentially damaging consequences of tanning [and skin bleaching] behaviours (e.g., skin cancer) has been found to be an ineffective intervention (Dennis et al., 2009), suggesting that appealing to an individual at the rational System 2 level via informational health promotion campaigns may not be effective. It may be more effective for interventions to appeal to the precognitive, System 1 processes. One way of doing this may be to use suitable images or visual interventions to reduce the risk of cognitively overloading the individual and activating System 2.

In terms of illness behaviours, visual appearance may contribute precognitively in the identification of illness in its early stages. For instance, pallor is known to be a predictor of illness (Hewson et al., 2000). However, recognition and perceptions of pallor may well be influenced by social and cultural factors. A study of the recognition of illness among almost 4000 young Kenyan children revealed that parents effectively used signs of their cultural concept of *del maratong* (a local term for 'pale-body/skin' which is not related to a specific anatomical site) rather than conventional anatomical pallor signs, to detect illness (Desai et al., 2002).

Visual signs may either *equate* to and be part of socially and culturally held definitions of health and illness *or* be seen as a *symbol* of underlying illness or health but without being heavily mediated at a conscious, cognitive level by any explicated or coherent set of beliefs. In other words, a parent may “know” that their child is ill without necessarily being aware why or being able to express how. This once again links back to the use of the ‘precognitive’ or System 1 cognitive processes: as there is minimal conscious mediation or rationalization occurring when the parent is observing and “knowing” that their child is ill, it is likely that they are using a combination of evolved cognitive heuristic processes and intuitive schemas, learned over past experience and through social knowledge. Thus, they are using System 1, precognitive processes to detect illness and will likely act on this information to decide upon the best course of action to take using the more rational System 2 processing (e.g., weigh up the pros and cons of attending a doctor versus home treatment, and select which is perceived to be the most effective health behaviour).

Moving beyond the precognitive, the importance of changes in images will now be considered. The discussion which follows summarises theory and literature mainly from psychology and medical sociology exploring the role of noticing changes in images as a mechanism to perceive symptoms. The section will discuss proposed mechanisms behind noticing and altering illness behaviours and proposed mechanisms underpinning misrepresentation/misinterpretation of changes and no behaviour change.

### Image changes as a prompt to symptom perception

#### Noticing changes and altering illness behaviours

Self-Regulatory Theories (e.g., Bandura, 1991; Baumeister et al., 1994) suggest that symptom interpretation and subsequent illness behaviours commence either when an individual notices a deviation in their established bodily norms, or when social messages from other people bring the deviation to their attention. This norm may be non-visible, such as in cases where there is pain or functional problems, in which case self-identification is more probable. However, for many clinical conditions, the deviation may be visual (e.g., skin colour, rash, bleeding, bruising). Due to their very public nature, visual cues are more likely to result in initial symptom perception stemming from social messages. If an illness is not causing discomfort, or if it has had a gradual onset, symptom perception is likely to begin with an individual or other noticing a visual or perceptible deviation from a norm. This act of noticing may not necessarily involve System 2 (slow, conscious) processes, but may instead reflect System 1 (fast, ‘automatic’) detection of difference that causes the visual sign to stand out and demand interpretation.

Established theory within the field of visual attention suggests that this act of noticing deviations from the norm may stem from an interplay between “goal-directed” and “stimulus-driven” factors (Corbetta & Shulman, 2002). Goal-directed factors are those which are noticed and interpreted in a ‘bottom-up’ cognitive manner: those factors which are directed and interpreted using existing knowledge and cognitive schema. Stimulus-driven factors, on the other hand, are those which draw our attention from the surrounding environment through sensory stimuli (e.g., vision, feeling, smell) and which are interpreted using more cognitively effortful ‘top-down’ processes. It has been well recorded that we are ‘set’ to selectively perceive, expect and infer aspects of goal-directed factors (e.g., Kalish & Lawson, 2007; Vernon, 1966); and thus we selectively perceive what is relevant to us, and interpret and generalise this knowledge in a way that is useful to us (Kalish & Lawson, 2007; Plous, 1993). For example, should an individual notice that a skin rash has occurred on their stomach (stimulus-directed factor), they may then proceed to hypothesise, given the location of the rash, that this may be serious, perhaps meningitis (goal-directed factor) based on their existing knowledge and may then choose to press a glass against the rash to see if it would disappear or seek medical assistance. Should the rash appear elsewhere, such as the arm, the individual may instead attribute causality to something less immediately worrisome, as they are not driven by a goal-directed, bottom-up, interpretive process.

It is evident that both the properties of the image/visual stimulus and the goals and expectations of the individual are important (Egeth & Yantis, 1997), and that these top-down and bottom-up processes interact to influence and individual’s health and illness behaviours. As discussed, in terms of the image, the important properties which will inform the individual in their health and illness behaviours are more complex than simple guidelines; there are some unified aspects but these are largely governed by the person’s own perceptions of their norms and their knowledge and past experiences. Some ‘universal’ elements for image properties in health and illness behaviours include the person noticing a deviation from their bodily norm, e.g., a skin rash); after noticing a process of evaluation occurs, whereby their personal norm is considered and the image is also compared to what the person knows about the general norm for the population and/or other similar conditions (e.g., other skin rashes that they have had, seen in others, or know of through education or through vicarious learning. The act of noticing can be through self-noticing or through socially transmitted messages, as previously discussed. Essentially, then, there are numerous levels to image noticing and interpretation, and the important properties in terms of enacting a change in health/illness behaviour will depend on the person’s own norms, their knowledge and importance placed on general norms, and social messaging around the image.

In terms of the individual, it is not only the conscious expectations and the act of noticing changes (e.g., Self-Regulatory behaviours; Bandura, 1991; Baumeister et al., 1994) which are important in health and illness behaviours, but also the unconscious processes (e.g., through System 1 processes; Kahneman, 2011).These unconscious processes may be heuristically governed, based on past experiences and pre-conceived expectations, which then go on to set current expectations and unconsciously ‘dismiss’ decision options for health/illness behaviours without the person being aware of this happening based on these past experiences and heuristics. These unconscious processes may also affect the information sought and afforded attention to in their decision making through the process of selective perception and selective attention, whereby people seek out and pay greater attention to information which conforms to their expectations and existing schema (Salemink et al., 2007; Sargent, 2007). The effect of unconscious decision making on health and illness behaviours are greatest with lay-people and with very experienced people: “When people have enough experience with a particular situation, they often see what they expect to see.” (Plous, 1993, p. 17). Both the properties of the image and/or the goals and expectations of the observer may therefore be important (Egeth & Yantis, 1997).

#### Misinterpreting changes and not altering illness behaviours

Symptom perception is a key element of illness behaviour research. To best understand illness behaviours and behaviour change, we must not only consider the mechanisms influenceing behaviour change (as in the former section), but also those theories and mechanisms explaining a lack of change in illness behaviours (or indeed detrimental illness behaviour change). This section will explore this latter theme.

Misperception or misinterpretation can lead to a failure to recognise important clinical signs, thus delay diagnosis. Further adding to complications around delay to treatment, gender differences exist in symptom perception, with females consistently being found to report more symptoms than males (van Wijk & Klok, 1997), skewing not only self-perception of symptoms, but also potentially the perceptions of importance attached to these by others. Approximately one-third of cancer patients have been found to delay seeking help for over three months (Mor et al., 1990). A significant contributory factor may be the misinterpretation or attribution of symptoms (Corner et al., 2005). However, research in this area has largely focused on delay between noticing a symptom and seeking help and treatment. The delay between the actual onset of a symptom and when it is first noticed may also be a major source of delay. Visual perception in both the initial noticing and subsequent interpretive processes is likely to be extremely important, but is currently largely over-looked.

Returning to the consideration of the evolved cognitive processes governed by System 1 decision making, acting on visual or other symptoms once these are perceived can be delayed further by misattribution of meaning and importance of these symptoms through the process of cognitive dissonance (Festinger, 1957, 1962). Cognitive dissonance theory suggests that when behaviours and beliefs are not congruent, we experience a ‘psychological discomfort’ and seek to reduce this discomfort. There are two ways in which to do this:

1. Changing behaviour or seeking help once symptoms are perceived; or
2. Changing or minimising attitudes towards the illness or help seeking (e.g., “it is not so serious”; “it will resolve itself”).

A classic example of attributing maladaptive meaning to a negative health behaviour, even when negative symptoms are perceived, is in tobacco smoking (McMaster & Lee, 1991). McMaster and Lee (1991) identified clear evidence of cognitive dissonance amongst smokers, with smokers knowing that they were at greater risk of health problems than non-smokers but rationalising that they were at less risk than their fellow smokers. This cognitive fallacy has clear implications, not for symptom perception per se, but for symptom interpretation and subsequent help seeking behaviour. The interpretation of symptoms and responses to these therefore involves a complex interplay of biopsychosocial factors. Understanding how this interplay impacts on and informs the perception and interpretation of symptoms is imperative for effective intervention development.

Further examination of the properties of images, the beliefs, cognitions and goals of observers, integrated within theories of visual attention, may prove helpful in understanding presentation delays and informing effective interventions. The importance of these issues has already been established in studies of health professionals’ detection of abnormalities in radiograph images (Manning et al., 2004), and that selective perception may play a role (Drew et al., 2013). However, their role in symptom detection among patients has been largely overlooked. It is therefore necessary to consider the role of images in symptom interpretation, and how this aligns to and influences conscious, cognitive processing in illness behaviours. This will now be discussed.

### The role of images in symptom interpretation: Embodied and influenced cognition

Although it is generally *assumed* that symptom interpretation cognitions are in verbal form, the theory in fact suggests that these are based on underlying abstract concepts. The nature of this form of abstraction is unknown, and potentially unknowable. However, it is possible that images can *embody* beliefs. For example, a patients’ drawing or even their mental represenattion of a broken arm may embody their beliefs in relation to its possible symptoms, cause, curability and general prognosis. There is recent empirical evidence that links aspects of patient’s drawing of their heart after a myocardial infarction to both subsequent behaviour and physical recovery (Broadbent et al., 2004, 2007). Images can elicit a real, emotional response both when viewed and when imagined or remembered. These emotive effects can become apparent with both concrete imagery (i.e., something “true to life” such as a spider or a house) and abstract imagery (e.g., a metaphorical or other representation of a concept or construct, such as an emotion). Evidence from cognitive psychology has suggested that image representation is dependent on retrieval from memory (Craik & Lockhart, 1972). In their ‘levels of processing’ framework, Craik and Lockhart (1972) posited that memory as a process, with fluidity across encoding and retrieval, constantly reinterpreting and adding meaning to past experiences through integrating new understandings to past experiences. The strength of a memory is considered to be increased when it has first been encoded strongly, and our image representations are strengthened when associated with either concrete imagery (Paivio, 1986) or high emotional arousal (Kensinger, 2009).

### The role of images in directly impacting emotions, affecting illness behaviour

Images may also generate a direct, unconscious emotional response independent of any cognition. For example, disgust, which is characterised by a desire to retreat from the stimulus, may be produced by the single sensory experience of viewing an image (Woody & Teachman, 2000). An example of an application of the use of negative emotion associated with images and health behaviours is in the treatment of phobias. Images feature prominently in phobias and anxiety disorders. Presentation of a still image of the feared stimuli can produce both an affective (anxiety, panic) and a biological (increased heartbeat, perspiration) response, even when there is no actual (real) object present. Spontaneous imagery is a common feature of anxiety disorders, and when mental representation of images is compared to verbal descriptions of the threatening stimuli, the imagery can produce higher levels of anxiety and a greater emotional response (Hirsch et al., 2006).

As images alone are capable of producing powerful emotional, cognitive, and associated biological responses, it is unsurprising that they may influence behaviour. Kleinman et al. (1978) reported the case of a 60-year-old woman, admitted to hospital and diagnosed as having ‘water in her lungs’. The woman proceeded to act bizarrely, repeatedly vomiting and urinating in her bed. It later transpired that this woman was the wife and daughter of plumbers, and believed the inner workings of the body to be akin to a system of inter-connected pipes. Her explanatory model/illness representation thus indicated quite logically to her that frequent expulsion of fluids from the body would cure the problem of water in her lungs. In this example the influence of her visual perception of the body as a system of pipes on her illness behaviour is clear. This is a relatively old example, but one which is helpful in providing clarity over the varied and anatomically incorrect representations that people may hold.

While there is some evidence for improved knowledge and anatomical accuracy in patients’ images in illness representations (Broadbent et al., 2019), there remains idiosyncratic differences in patient’s drawings of their illnesses which may help to predict their health outcomes (Broadbent et al., 2019; Petrie & Weinman, 2012). For example, Petrie and Weinman (2012) describe the drawings made at three timepoints by two patients who had underwent heart bypass surgery; while both patients drew increasingly ‘healthy/healing’ pictures of hearts, one also focused also on the heart rhythm improving, which Petrie and Weinman (2012) highlight is an important indicator for recovery. In their systematic review of 101 papers published exploring patient drawings of illnesses, Broadbent et al. (2019) found evidence indicating that where patients had drawn larger images and more organ damage, outcomes and health perceptions were worse. Improvements to patient-practitioner understandings of the patient’s image representations of their illness may therefore be helpful in improving outcomes and targeting psychoeducation where required to help improve illness behaviours related to recovery (Petrie & Weinman, 2012).

Although there is evidence to support the use of images and imagery for behaviour change, there has been relatively little *consistent* attention in health research; with the majority of the research being confined almost exclusively to the investigation of children’s views concerning illness (Guillemin, 2004). However, the investigation of adults’ mental image representations of illness is an exciting emerging field of research, with advocates of its value scattered across several disciplines (e.g., Broadbent et al., 2004; Cross et al., 2006; Guillemin, 2004).

Due to the infancy of the work in this area, there are several important and fundamental questions which have not yet been addressed:

1. To what degree does the experience of illness encompass images, for whom, and during which illnesses?
2. How do images relate to current theories, such as illness representation and explanatory models? And are these embedded within the models or do they exist independently?
3. What are the relationships between cognitions, images, and emotions?
4. Do images produce or influence cognitive processes, or do cognitive processes assist and change the rudimentary image into a more concrete image?

It is also important to consider the theories and literature on images in illness behaviours discussed in relation to health care: how do images affect the experience of illness, the behaviours associated with it, and how might they affect health outcomes? The proceeding section will explore these issues.

## The importance of images in health care

The importance of researching and accessing patients’ mental representations of images is three-fold. These may influence illness experience, illness behaviour, and – directly and indirectly – health outcomes. As illness experience and illness behaviours have been covered to some degree in the previous sections of the current paper, these sections will be comparatively brief; only covering health care-focused applications. The section on health outcomes will be longer, as we have not covered this in as great depth so far within the current paper.

### Illness experience

An individual’s experience of illness symptoms can be influenced by their image representations associated with the symptoms. To illustrate this, consider the case of indigestion versus tapeworm. A person experiencing a mild but persistent stomach upset, eventually consults a doctor to be told that it is: a) mild indigestion; or b) a tapeworm - an infestation of the digestive tract with the tapeworm parasite. This is a ribbon shaped creature which can come from a cow, pig, sheep, dog, cat and other animals. Tapeworm eggs are passed in the stools of a person who is infected and spread through water or surfaces contaminated with faeces. Despite both conditions presenting identical symptoms, the images associated with the latter elicits entirely different cognitions and emotions than those that would be associated with the more common condition of indigestion. The two conditions are therefore experienced in very different ways, despite the physiological symptoms being similar.

### Illness behaviour

Image representations can also affect an individual’s illness behaviours. For example, Williams et al. (2007) examined adherence to non-medicinal treatments in children with cystic fibrosis, and found that their visualisation of mucus building up in their lungs, rather than any verbalised beliefs, was the chief motivator for them to perform chest physiotherapy. Similarly, Kleinman et al. (1978)afore-mentioned case study of the woman who was admitted to hospital with ‘water in her lungs’ and who had attempted to cure this through repeated vomiting and urination clearly illustrates that visualisation can influence illness behaviours, as the patient’s image of the body as a system of interconnected pipes dictated her behaviour as she attempted to cure her illness (i.e., excessive expulsion of liquids).

### Health outcomes

Health outcomes have also been shown to be influenced by patients’ image representations of their conditions. Broadbent et al. (2004) asked patients who had suffered a myocardial infarction (MI) to draw their hearts in a pre- and post-MI state. This study found that the patients’ drawings of their heart pre- and post- MI were not only accurate indicators of underlying beliefs about their heart attack, but also a better predictive marker of time taken to return to work than any clinical indicator. Those who perceived less damage to their heart recovered more quickly, regardless of the extent of actual damage.

The need to address patients’ images is also important due to the potential for inaccuracy. Inaccurate images nourish inaccurate and potentially damaging misunderstandings of illness. The breadth of inaccuracy in lay understandings of anatomy was demonstrated by Boyle (1970) in his comparison of doctors’ and patients’ location of various organs within a human silhouette. In his study, Boyle (1970) asked patients and doctors to identify within a picture of a human silhouette where the kidneys and heart were located. Surprisingly, the identified locations of these organs were widely varied; clearly indicating that we cannot assume that lay members of the population have a strong understanding and knowledge about their internal bodily functioning or even the positioning of major organs.

External images pertaining to illnesses and the body undoubtedly play a role in subjective visualisations of illness. In clinical settings patients observe images informally in the form of sketches during consultations, and formally through use of x-rays, ultrasounds, and endoscopic cameras. Visual language or metaphors used by health care professionals in interactions with patients may also have a significant impact (Harrow et al., 2008).

The health service in the UK readily and increasingly employs imagery of the body in its growing number of health promotion campaigns, but their use is largely atheoretical (Williams & Cameron, 2009), despite a wealth of theoretical models attempting to define and explain health and illness behaviours existing in the health, psychology and sociology literature (e.g., Theory of Planned Behaviour, Leventhal’s Self-Regulatory Model, Health Belief Model). This is in a context of increasing emphasis on shared decision-making, and a post-modern culture whose population’s everyday lives are increasingly dominated by the visual and who interpret the world and find meaning in more visual ways (Mirzoeff, 1999). Within this visually-focussed society, Government spending on health promotion campaigns involving visual media will almost certainly increase. A strong theoretical premise to justify additional spending and to maximise the effectiveness of health promotion campaigns is therefore crucial.

The backbone of verbal and textual health interventions is usually the spectrum of behavioural theory, but the applicability of these theories to image based interventions is unknown. The lack of image-appropriate theory is evident in the fact that the UK’s Department of Health consulted the public via a purpose-created website, over which graphic images would encourage smoking cessation if introduced onto cigarette packets. The consultation followed evidence from Canada indicating the potential effectiveness of pictorial warnings (Department of Health, 2006), though the intricacies of their impact – how, why, which features – have yet to be determined.

Notwithstanding their atheoretical nature, in the broader health context it has been established that image based interventions can be effective. Petrie et al. (2002) carried out a Randomised Control Trial of an intervention for MI patients, using drawings to explain pathophysiology and symptoms and addressing patients’ misconceptions. Compared to controls (who received standard MI educational materials), the intervention group reported increased preparedness to leave hospital, significantly fewer angina symptoms at three-month follow-up, and they returned to work significantly faster after their MI. To further illustrate, Shahab et al. (2007) carried out a Randomised Control Trial investigating an image based intervention to encourage smoking cessation. Smokers in the intervention group received an ultrasound photograph of their own artery showing atherosclerotic plaque alongside an image of a healthy artery, while controls received verbal information standard to the clinic’s normal practice. The intervention group showed increased perceived susceptibility to atherosclerosis, higher rates of engagement in smoking cessation behaviours, and reported increased intention to stop smoking when followed up at four weeks; although the latter effect was only reported by those with higher levels of self-efficacy. An earlier, similar study by Bovet et al. (2002) employed the same technique with a sample of 153 smokers and found an increased quit-rate at six-months among the group which received ultrasound photographs of their plaques, compared to controls. These findings demonstrate the potential for effective image based interventions in improving health behaviours.

To improve these interventions, inform future development, and target them appropriately, an underpinning theory is required. Of primary concern is the exposure of the complex inter-relationships between the following variables: socioeconomic status and gender, culture, image representations, external images, illness perceptions, cognitions, emotions, and behaviour.

## Potential theoretical explanations

This final section will discuss two of the most clearly aligned existing theories which may be relevant in helping explore these inter-relationships. First, illness coherence. This is the extent to which an individual feels they have a clear understanding about a threat to their health and how the recommended action will reduce it. The role of illness coherence was found to be crucial in promoting smoking cessation among females whilst educating them about the associated risk of cervical cancer (Hall et al., 2004). Coherent understandings need not be biologically plausible; simply plausible to the individual based on their subjective understanding of the body and how it works. For instance, if a patient believes that tooth plaque is composed of germs that are killed by toothpaste, and brushing behaviour increases because of this, then positive health behaviour results despite the less than accurate associated understanding. Conversely, an obese patient presenting with knee pain may not believe that their pain and weight are related and may therefore disregard a doctor’s suggestion of losing weight to alleviate their pain.

The second potential theoretical explanation may be drawn from the broad range of psychological processes discussed in the current paper; in particular those focused on cognitive processing. Through understanding the previously discussed underlying psychological mechanisms underpinning health and illness behaviours (such as the dual process theories, exploitation of System 1 decisional processing and quasirationality models, concrete and abstract image representation, and encoding/retrieval from memory), existing theoretical explanations attempting to draw together the inter-related elements affecting health and illness behaviours (socioeconomic status and gender, culture, image representations, external images, illness perceptions, cognitions, emotions, and behaviour) can be better informed and strengthened.

Within positivist and post-positivist traditions that employ quantitative methods, a focus on the verbal may stem from a practical assumption that the commonality of meaning for words may be assessed and thus counted (Weinman et al., 1996). Within interpretivist paradigms that support the majority of contemporary qualitative research there *is* an acknowledgement of the ways in which beliefs and meanings may be embodied in forms other than words. However, almost all qualitative research continues to focus exclusively on words. Lessons for developing better research understanding images and their representations in health and illness behaviours can, however, be learned.

By not researching this area we may be: failing to address an important dimension of people’s illness experience; using images both formally and informally in ineffective, inefficient or even unhelpful ways; and wasting an opportunity to use an increasingly acceptable and powerful tool to influence experience, behaviour, and health outcome. Descriptive primary and inductive research is required to address some of the fundamental questions proposed in this paper and these might best begin by examining patient experience.

## Conclusion

Illness behaviours are complex but are well studied across the social and behavioural science disciplines. The literature across these disciplines demonstrates convergence across four key areas, despite being theoretically and philosophically contrasting. As discussed, the first commonality which these fields emphasise the importance of understanding the importance of patient perspective, emphasising illness beliefs, explanatory models, or illness representations. The second convergence is around recognising the person’s perception of the cause, their sense of identity with the illness, the consequences, and the level of control that they have or believes that they have over their health outcomes. Third, the recognition across the disciplines that health beliefs may influence illness experience, behaviours, and outcomes; and, finally, the cross-discipline emphasis on understanding illness beliefs and experience has to date been achieved through an almost exclusive focus on the written or spoken word. The current paper has focused upon this fourth commonality, exploring current theoretical and empirical evidence across the social and behavioural sciences relating to the role of images in illness behaviours. Images in the context of the current paper are considered to be both visual and mental image representations. The core findings from the current paper indicate that images can affect illness behaviours in multiple ways, at both an unconscious/precognitive and a conscious level. The use of images in healthcare can elucidate a person’s and practitioner’s understandings about an illness, can facilitate communication about the illness and treatment decision making, and may subsequently support better health outcomes if effectively applied. It is this latter point which public health practitioners, academics, and health professionals could further develop to best support health outcome improvement for patients.

The decision to focus on the role of images in understanding illness behaviours in the current paper was taken to due to the relative scarcity of cross-disciplinary theoretical discussion first, and, second, that the existent theoretical underpinnings are largely not applied to visual intervention development in current practice. In recent years, there has been an expansion on the number and nature of health promotion interventions, and in particular on using image-based representations. While there has been research across disciplines on illness representations and behaviours and their relation to patients’ image-based understandings at both a conscious and unconscious level, the evidence base for applying this theory and literature to visual/image-based intervention development is scarce. It appears that the majority of visual and image-based interventions are not designed using an explicit theory-informed approach. Based on the evidence discussed in the current paper, we argue that through not integrating theoretical underpinnings and understandings of image-based health and illness representations into visual health interventions, their efficacy and effectiveness will be limited at best and ineffective at worst, leading to research waste. Future academics, health professionals, and other stakeholders who wish to develop visual interventions should engage with the literature across fields and use this to develop an explicit and clear theoretical underpinning framework for their intervention to improve the quality and effectiveness. Ideally, interdisciplinary working should be employed to bring together fields of expertise, to lead to more efficacious visual interventions being developed.

## Author Biographies

**Brian Williams**: Professor Williams has over 25 years research experience and 12 years’ experience of leading two different joint university multidisciplinary research units. Although he maintains an international research profile in behavioural science and research methods, his key focus and national and international reputation is on development of research expertise at the individual, group and organizational level – especially in the area of nurses, midwives and allied health professionals. This includes levering and facilitating national policy, and working across HEIs.

**Jennifer Murray**: is an active researcher in the areas of forensic psychology and applied health research, with an overarching theme of decision science pulling these two areas together. She is passionate about developing useful, theoretically sound interventions and outputs from her research and typically collaborates across multidisciplinary teams and non-academic stakeholders to make her research as applicable to ‘real practice’ as possible. The key focus of Murray's work is on developing clinically useful research which can inform and/or support day to day practice.
